# JOURNAL OF GERONTOLOGY: PSYCHOLOGICAL SCIENCES

**DOI:** 10.1093/geroni/igae098.0908

**Published:** 2024-12-31

**Authors:** Duke Han

**Affiliations:** University of Southern California, Los Angeles, California, United States

## Abstract

N/A

